# Allelopathic Effects of Corn Straw and Its Water Extracts on Four Weed Species and Foxtail Millet

**DOI:** 10.3390/plants13101315

**Published:** 2024-05-10

**Authors:** Shuqi Dong, Jiaxin Dong, Peiyao Li, Bo Cao, Mengyao Liu, Zhenyu Guo, Xie Song, Yongqing Ma, Chunyan Hu, Xiangyang Yuan

**Affiliations:** 1 College of Agriculture, Shanxi Agricultural University, Jinzhong 030800, China; dongshuqi@sxau.edu.cn (S.D.); djx199907@163.com (J.D.); peiyaoli666@163.com (P.L.); caobo000315@163.com (B.C.); 15903556943@163.com (M.L.); sxndsxe@163.com (X.S.); 2 College of Plant Protection, Shanxi Agricultural University, Jinzhong 030800, China; gzy2816@163.com; 3 Institute of Soil and Water Conservation, CAS & MWR, Yangling 712100, China; mayongqing@ms.iswc.ac.cn

**Keywords:** corn straw, water extract, allelopathy, weeds control

## Abstract

Straw covering is a protective tillage measure in agricultural production, but there is relatively little research on the allelopathic effects of corn straw on weeds and foxtail millet. This experiment studied the allelopathic effects of corn straw on four weeds (*Chenopodium album*, *Setaria viridis*, *Echinochloa crus-galli* and *Amaranthus retroflexus*) in foxtail millet fields, and also measured the growth indicators of foxtail millet. The study consisted of Petri dish and field experiments. Five treatments were used in the Petri dish experiment: clear water as control (0 g/L, TCK) and four types of corn straw water extracts. They were, respectively, the stock solution (100 g/L, T1), 10 X dilution (10 g/L, T2), 50 X dilution (2 g/L, T3), and 100 X dilution (1 g/L, T4) of corn straw water extracts. Additionally, seven treatments were set up in the field experiment, consisting of three corn straw covering treatments, with covering amounts of 3000 (Z1), 6000 (Z2) and 12,000 kg/ha (Z3), and four control treatments—one treatment with no corn straw cover (CK) and three treatments involving the use of a black film to create the same shading area as the corn straw covered area, with black film coverage areas of 50% (PZ1), 70% (PZ2), and 100% (PZ3), respectively. The results showed that the corn straw water extract reduced the germination rate of the seeds of the four weeds. The T1 treatment resulted in the allelopathic promotion of *C. album* growth but the inhibition of *S. viridis*, *E. crus-galli*, and *A. retroflexus* growth. Treatments T2, T3, and T4 all induced the allelopathic promotion of the growth of the four weeds. The order of the effects of the corn straw water extracts on the comprehensive allelopathy index of the four weed seeds was as follows: *C. album* > *S. viridis* > *A. retroflexus* > *E. crus-galli*. With an increase in the corn straw mulching amount, the density and total coverage of the four weeds showed a gradual downward trend, whereas the plant control effect and fresh weight control effect showed a gradual upward trend. All indices showed the best results under 12,000 kg/ha of mulching and returning to the field. Overall, corn straw coverage significantly impacted the net photosynthetic rate and transpiration rate of foxtail millet and increased the yield of foxtail millet. Under coverages of 6000 and 12,000 kg/ha, the growth of foxtail millet is better. Based on our findings, we recommend a corn straw coverage of 12,000 kg/ha for the allelopathic control of weeds in foxtail millet fields.

## 1. Introduction

Foxtail millet (*Setaria italica* (L.) Beauv) is an annual gramineous plant that originated in China [1,2] and has a short growth period, rich nutritional value [3,4,5], and strong drought and salt stress resistance. It is the main miscellaneous grain crop in dry farming ecological agriculture [6,7] and can be used as a substitute crop to supplement other economically valuable crops [8]. After years of development, the foxtail millet industry has made continuous progress under the premise of green and sustainable ecological development. In recent years, with the support of the government and promotion of the market, foxtail millet industrialization has accelerated. Foxtail millet is widely cultivated across northern China [9]. With the structural adjustment of the agricultural industry and the increased demand for miscellaneous grains, the yield of foxtail millet has shown a gradual upward trend. However, many types of weeds are found in foxtail millet fields that have strong stress resistance and are difficult to control, and few herbicides are suitable for foxtail millet fields [10]. Currently, only seven herbicides are registered with the China Pesticide Information Network. Foxtail millet is very sensitive to chemical herbicides [11]. Therefore, manual weeding remains the main method of weed control during the growth period of foxtail millet, which is laborious.

Straw is an important byproduct of crop production and a valuable renewable energy source. After straw covering, chemical substances are released through pathways such as root secretion, stem and leaf volatilization, rain and fog leaching, and plant residue decomposition, which affect the surrounding plants or microorganisms [12,13,14]. These substances are called allelopathic substances and are secondary metabolites of diverse types with a small molecular weight and simple structure. They affect the growth of neighboring crops, target crops, and weeds through allelopathic effects [15,16]. The majority of discovered allelochemicals are terpenes, phenols, and organic acids. These allelopathic substances can exhibit direct or indirect inhibitory or promoting effects on different receptor plants or microorganisms [17].

Straw covering can effectively reduce soil moisture evaporation and regulate soil temperature [18,19,20]. Microbial decomposition and rainwater leaching covered with straw can improve soil fertility [21] and physicochemical properties [22,23,24], can promote soil water fertilizer interaction, and are widely used in conservation tillage [25,26]. Cai et al. [27] revealed that the soil water storage capacity of corn straw covered within a soil depth of 200 cm increased. Additionally, grain yield showed an increase with the increase in straw coverage, improving water use efficiency. Valvi et al. [28] showed that the water extract of teak leaves had an inhibitory effect on rice germination and other growth parameters, which was proportional to the concentration of the extract; the higher the concentration, the stronger the inhibitory effect. Akhtar et al. [29] showed that corn straw mulching reduces the demand for nitrogen fertilizer for wheat by regulating soil moisture and temperature and improves the nitrogen use efficiency, yield, and water use efficiency of wheat. Crawford et al. [30] confirmed that the straw mulching of cereal crops inhibited the growth of weed seedlings in edamame fields and reduced the emergence rate of weeds in edamame fields by 20% whilst not interfering with the growth of soybean. Akhtar et al. [31] found that wheat straw mulching significantly improved photosynthesis, SPAD value, growth, biomass, and the seed yield of soybeans. Zhu et al. [32] found that the biomass of *E. crus-galli* covered with 1, 3, 5, 7, and 9 cm rapeseed straw decreased by 65.74%, 80.18%, 81.15%, 70.99%, 55.65%, and 27.22%, respectively. Mehra et al. [33] illustrated that the severity of wheat diseases varies depending on residual coverage rate. Disease severity increased nonlinearly with the residual level increase, and residual coverage had a significant effect on disease severity in all treatments.

Corn is the predominant crop grown in China. However, there are few reports on the allelopathic effects of corn straw on weeds in foxtail millet fields. In this study, using a combination of Petri dish and field experiments, the biological indices of four weeds in foxtail millet fields were measured in a Petri dish experiment and after corn straw mulching, and the number of weeds was investigated in a field experiment. Additionally, we analyzed the growth and physiological indicators of foxtail millet and the allelopathic effects of corn straw on foxtail millet and its associated weeds. These findings provide a scientific reference for the application of corn straw in foxtail millet field mulching.

## 2. Results

### 2.1. Effects of Corn Straw Water Extracts on Four Weed Species

**Figure 1:**
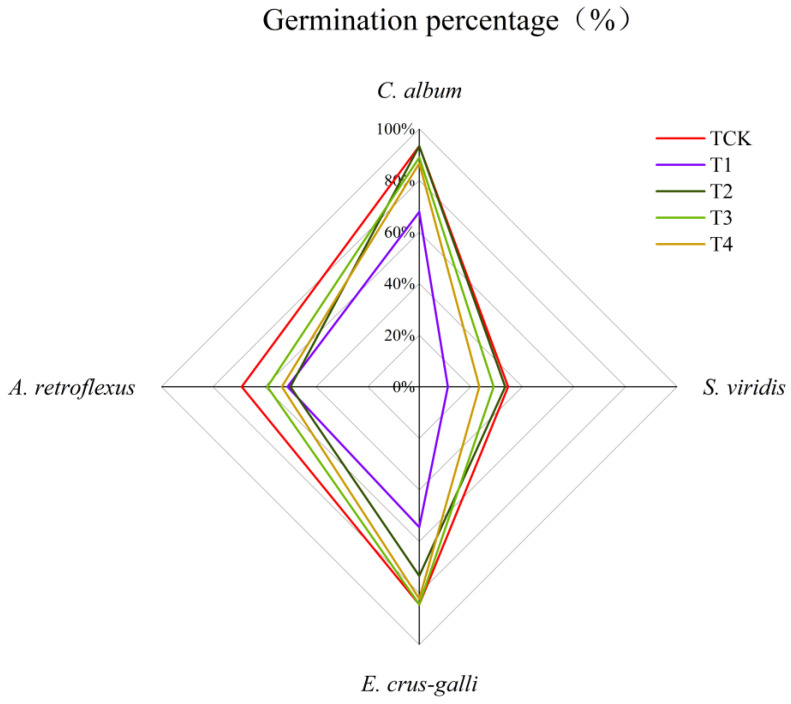
Effect of corn straw water extract on seeds germination rate of the four weeds (Petri dish experiment). TCK—Clear water control; T1—the stock solution of corn straw water extracts; T2—10× dilution of corn straw water extracts; T3—50× dilution of corn straw water extracts; T4—100× dilution of corn straw water extracts.

All the concentrations of corn straw water extract reduced the germination rate of the seeds of the four weeds. Compared with that of TCK, the T4 treatment significantly reduced the germination rate of *C. album* and *A. retroflexus*. The seed germination rates of *C. album*, *S. viridis*, and *E. crus-galli* was lowest under the T1 treatment, which significantly decreased by only 25.55%, 23.33% and 30.00%, respectively, compared with that of TCK, whilst the seed germination rates of all four weeds were significantly reduced compared with that of TCK. The T2 treatment significantly reduced the germination rates of *E. crus-galli* and *A. retroflexus* by 11.11% and 18.89%, respectively, compared with that of TCK. There was no significant difference in the germination rates of the four weed seeds between the T3 treatment and TCK groups (Figure 1).

**Figure 2:**
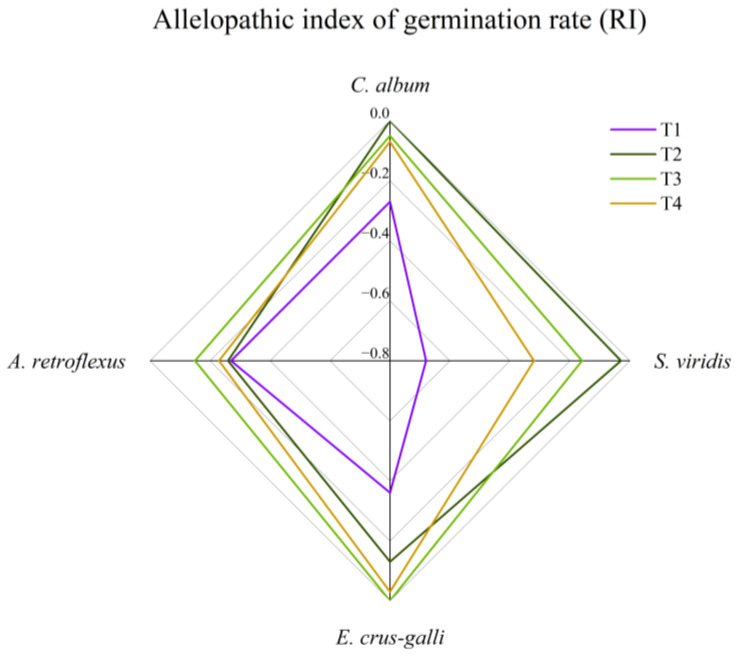
Effect of corn straw water extract on the allelopathy index of seeds germination rate (Petri dish experiment).

Different concentrations of corn straw water extract had different allelopathic effects on the germination rates of the seeds of the four weeds, but all the treatments showed the allelopathic inhibition of germination. Under the T1 treatment, the allelopathic index of the germination rate of the four weeds was the largest and the allelopathic inhibition effect was the strongest, compared to that of the other treatments. The order of the inhibition effect of T1 on the seed germination rates of the four weeds from strong to weak is *S. viridis* > *E. crus-galli* > *C. album* > *A. retroflexus* (Figure 2).

**Figure 3:**
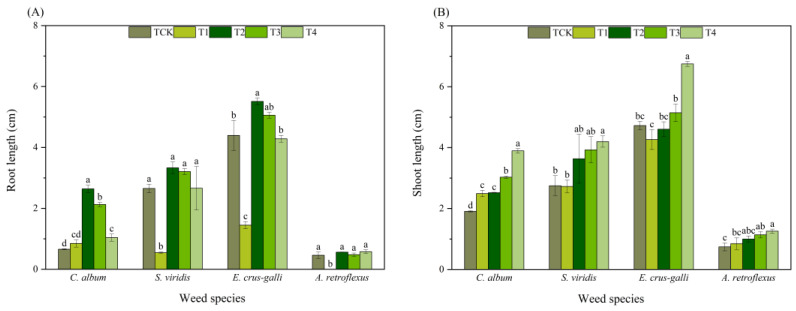
Effects of corn straw water extract on root length (**A**) and shoot length (**B**) (Petri dish experiment). Error bars indicate the standard error of means. Note: Comparison between treatments of different concentrations on the same weed species, with lowercase letters representing a significant difference (*p* < 0.05).

The T1 treatment allelopathically inhibited the root lengths of *S. viridis* and *E. crus-galli* by 79.48% and 67.06%, respectively, and the shoot lengths by 0.85% and 9.74%, respectively, compared with that of TCK. Thus, the inhibition effect on the root length was stronger than the effect on the shoot length (Figure 3). However, the T1 treatment increased the root and shoot length of *C. album* through allelopathy. The root length of *A. retroflexus* seeds was inhibited by allelopathy, and the shoot length of *A. retroflexus* seeds was promoted by allelopathy. The T2, T3, and T4 treatments promoted the root and shoot lengths of the four weeds, and the allelopathic-induced reduction in the shoot length by the T4 treatment was significantly different from that of the TCK treatments. With an increase in the concentration of the corn straw water extract, the increase in root length gradually decreased, while shoot length gradually increased (Figure 3).

**Figure 4:**
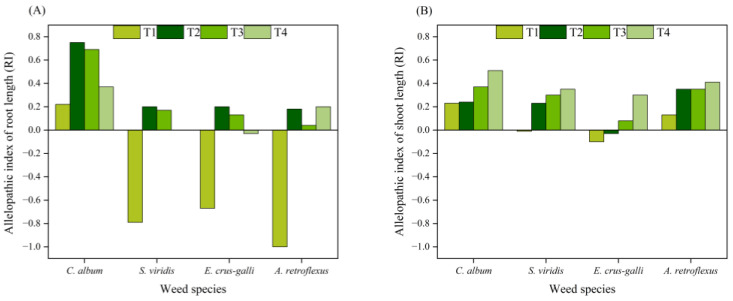
Effects of corn straw water extract on allelopathic index of root (**A**) and shoot (**B**) length (Petri dish experiment).

The different corn straw water extract treatments had different allelopathic effects on the root and shoot lengths of the four weeds. The T1 treatment induced allelopathic reductions in the root and shoot lengths of *S. viridis* and *E. crus-galli* and had the strongest allelopathic effect on the root lengths of *A. retroflexus*. Under the T2, T3, and T4 treatments, allelopathy resulted in a gradual decrease in root length with increased dilution ratios of corn straw water extract, whilst shoot length gradually increased with increased dilution ratios of corn straw water extract. The T4 treatment had the greatest effect on the allelopathy index of the shoot lengths of the four weeds, which were 0.51, 0.35, 0.30, and 0.41 (Figure 4).

**Figure 5:**
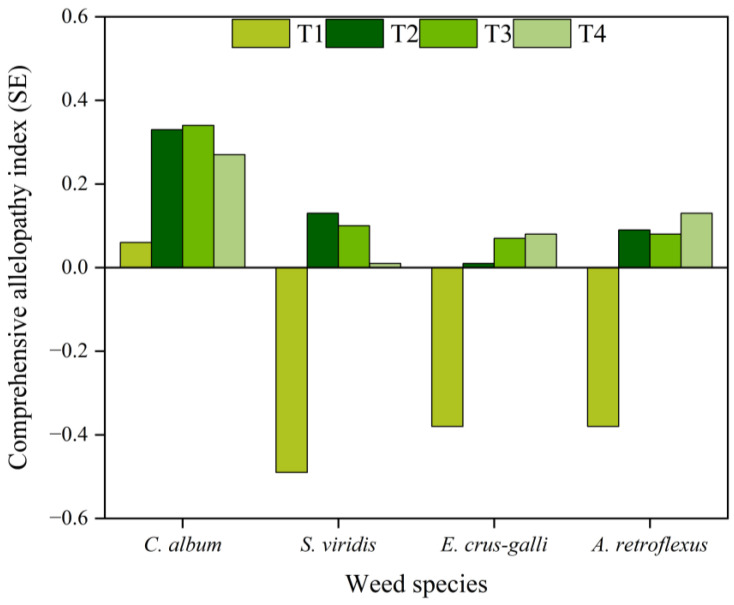
Effect of straw water extract on the comprehensive allelopathic index of the four weeds seeds (Petri dish experiment).

The comprehensive allelopathy index of the corn straw water extracts for the four weeds significantly differed, and the comprehensive allelopathic effects on *C. album* were the greatest. The T1 treatment resulted in the allelopathic promotion of *C. album* and allelopathic inhibition of *S. viridis*, *E. crus-galli* and *A. retroflexus*. In contrast, the T2, T3, and T4 treatments all induced the allelopathic promotion of the growth of the four weeds. With the increasing dilution ratios of the corn straw water extract, the comprehensive allelopathic effect of the extracts on *C. album* and *S. viridis* gradually decreased, whilst the comprehensive allelopathy effect of the extracts on *E. crus-galli* and *A. retroflexus* gradually increased. The order of the effects of the corn straw water extracts on the comprehensive allelopathy index of the four weeds from strong to weak was *C. album* > *S. viridis* > *A. retroflexus* > *E. crus-galli* (Figure 5).

### 2.2. Effects of Corn Straw Mulching on the Four Weeds

Under different corn straw mulching amounts, the number of weeds under the Z3 treatment was the lowest after rainfall. With the increase in corn straw mulching, the density of weeds gradually decreased, and with increasing incidences of rainfall, the reduction in weed density due to corn straw mulching gradually decreased. Under the Z2, and Z3 treatments, after each rainfall event, the densities of *C. album* and *E. crus-galli* were significantly lower than those of CK, which has an obvious allelopathy inhibition effect. After the third rainfall, the density of *A. retroflexus* under Z3 treatment was significantly lower than that under CK treatment (Table 1).

Five days after the first rainfall event, the densities of the four weeds under the Z1, Z2, and Z3 treatments were lower than that under CK, but there was no significant difference between the Z1 and PZ1 treatments. Under the cover of Z2 straw, *C. album* and *A. retroflexus* were significantly different from the PZ2, but *S. viridis* and *E. crus-galli* were not significantly different from the PZ2. Therefore, 5 days after the first rainfall, the reduction in weed density in the Z1 and Z2 treatments primarily depended on the physical coverage provided by the straw (Table 1).

Five days after the second rainfall event, the densities of *C. album* and *E. crus-galli* under the Z1, Z2, and Z3 treatments were significantly lower than those under CK. However, the density of *S. viridis* under the Z1 treatment was significantly higher than that under CK. The density of the four weeds under the Z2 treatment were lower than those under CK. *E. crus-galli* and *A. retroflexus* showed no significant difference between the Z1 and PZ1 treatments. *S. viridis, E. crus-galli* and *A. retroflexus* showed no significant difference between the Z2 and PZ2 treatments. Therefore, the decrease in the prevalence of the four weeds under the Z1 and Z2 treatments primarily depended on the physical covering effect of the straw 5 days after the second rainfall (Table 1).

Five days after the third rainfall event, the densities of the four weeds were significantly different between the Z1 and PZ1 treatments or between the Z2 and PZ2 treatments except for *A. retroflexus*. The Z3 treatment effectively reduced the prevalence of the four weeds, which was the result of the physical covering effect of the straw and allelochemicals (Table 1).

**Figure 6:**
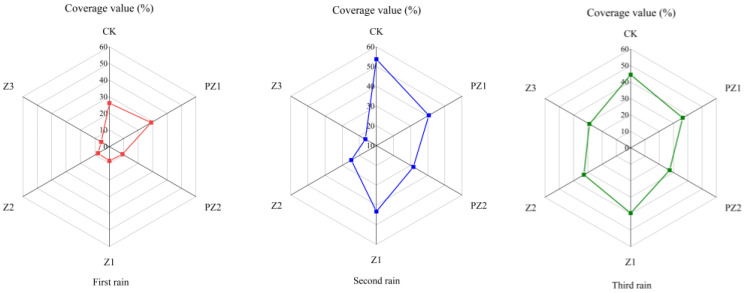
Total weed coverage value under different corn straw mulching (field experiments). No weed growth under PZ3 treatment.

The Z1, Z2, and Z3 treatments significantly reduced the total coverage of weeds in foxtail millet fields compared with the coverage in the CK group, and with increased corn straw coverage, the total coverage of weeds gradually decreased. Total weed coverage under the Z3 treatment was consistently lower than that under the Z1 and Z2 treatments (Figure 6).

Five days after the first rainfall, the total weed coverage under the Z1, Z2, and Z3 treatments was significantly lower than that of CK, and total weed coverage was lowest under the Z3 treatment. There was no significant difference in weed coverage between the Z1 and PZ1 treatments five days after the second and third rainfall events. Therefore, the reduction in weed coverage under the Z1 treatment five days after the first rainfall was the result of both the physical covering effect of the straw and allelochemicals, and the reduction in weed coverage five days after the second and third rainfall events primarily depended on the physical covering effect of the straw. There was no significant difference in weed coverage between the Z2 and PZ2 treatments five days after the first and second rainfall events. Total weed coverage under the Z2 treatment was significantly higher than that under the PZ2 treatment five days after the third rainfall event. Therefore, the decrease in total weed coverage under the Z2 treatment five days after the first and second rainfall events was due to the combined effect of the physical covering of the corn straw, whilst the decrease in total weed coverage five days after the third rainfall event primarily depended on the physical covering effect of corn straw and allelochemicals (Figure 6).

With increasing corn straw mulching amounts, the number and fresh weight of weeds gradually decreased, while the plant control effect and fresh weight control effect gradually increased. With increased rainfall incidences, the number and fresh weight of weeds gradually increased, while the plant control effect of and fresh weight control effect of weeds gradually decreased (Table 2).

Five days after the first rainfall, the plant control effect and fresh weight control effect under the Z3 treatment were the highest, reaching 74.38% and 61.03%, and the number and fresh weight of weeds under the Z1, Z2, and Z3 treatments were significantly lower than those of CK. Compared with that of PZ2, the control effect under the Z2 treatment increased by 14.63%. Five days after the second rainfall, the number and fresh weight of weeds treated with Z3 were significantly lower than those treated with CK, whilst plant control effect and fresh weight control effect were significantly higher than those under the Z1 and Z2 treatments. Under the Z1 and Z2 treatments, the plant control effect and fresh weight control effect were lower than those under PZ1 and PZ2 treatments. Five days after the third rainfall event, the number and fresh weight of weeds under the Z2 and Z3 treatments were significantly different from those of CK, while the plant control effect and fresh weight control effect of Z3 treatment were significantly higher than those of Z1 and Z2 (Table 2).

### 2.3. Effects of Corn Straw Mulching on Foxtail Millet

#### 2.3.1. Effects of Corn Straw Mulching on Plant Height, Leaf Area and SPAD of Foxtail Millet

**Figure 7:**
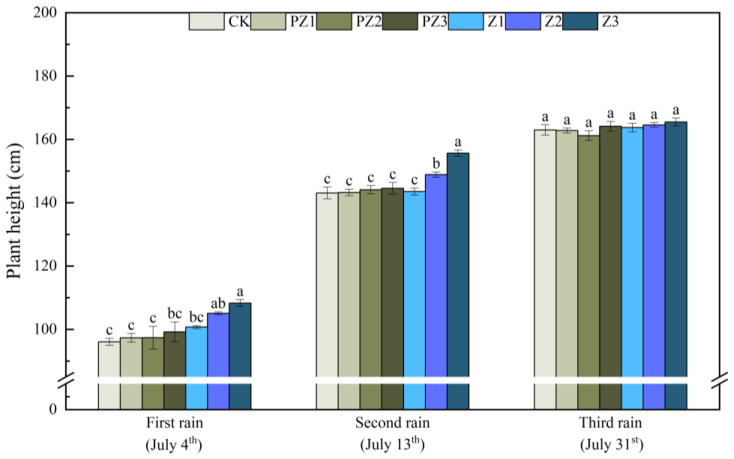
Effect of corn straw mulching on foxtail millet plant height (field experiments). Note: Comparison between treatments of different mulching on the same days, with lowercase letters representing a significant difference (*p* < 0.05).

**Figure 8:**
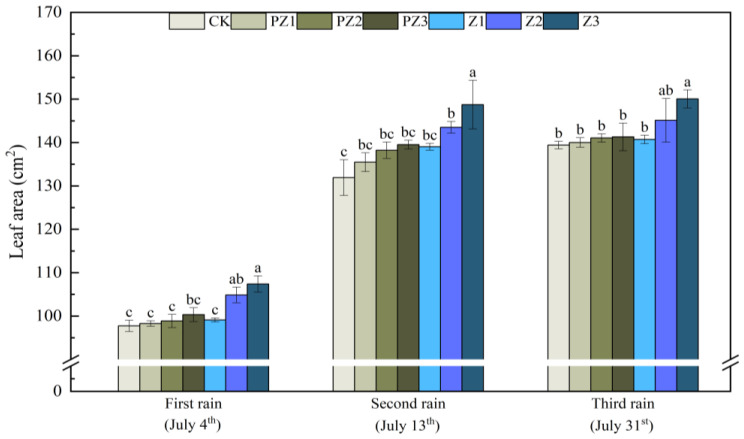
Effect of corn straw mulching on foxtail millet leaf area (field experiments). Note: Comparison between treatments of different mulching on the same days, with lowercase letters representing a significant difference (*p* < 0.05).

**Figure 9:**
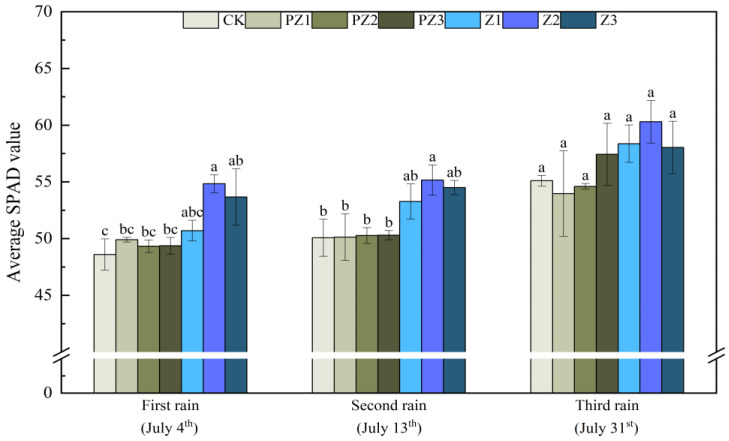
Effect of corn straw mulching on soil plant analysis development SPAD value of foxtail millet (field experiments). Note: Comparison between treatments of different mulching on the same days, with lowercase letters representing a significant difference (*p* < 0.05).

The plant height, leaf area, and SPAD value of foxtail millet all improved under the varying coverages of corn straw. Specifically, as corn straw coverage increased, the plant height and leaf area of foxtail millet gradually increased, while the SPAD value first increased and then decreased. Following the first rainfall that lasted for 5 days, the plant height, leaf area, and SPAD value of Z1 treatment increased by 4.67 cm, 1.36 cm^2^, and 2.1, respectively, compared with CK; however, this difference was not significant. Compared with CK, the plant height, leaf area, and SPAD value of Z2 treatment increased by 9 cm, 7.12 cm^2^, and 6.23, respectively, with this difference not being significant. Compared with CK, the plant height and leaf area of foxtail millet treated with Z3 increased by 12.33 cm and 9.65 cm^2^, respectively, with these differences being significant. Additionally, the SPAD value increased by 5.07; however, this increase was not significant. The plant height, leaf area, and SPAD value of foxtail millet all increased under different corn straw mulching amounts compared with the corresponding area of black film mulching. The plant height, leaf area, and SPAD value of foxtail millet in Z1 treatment increased by 3.4 cm, 0.82 cm^2^, and 0.8, compared with PZ1 treatment, although the differences were not significant. Compared with PZ2 treatment, the plant height of foxtail millet in Z2 treatment increased by 7.7 cm; however, the difference was not significant. The leaf area and SPAD value increased by 5.99 cm^2^ and 5.5, with significant differences. Compared with PZ3 treatment, the plant height and leaf surface integral of foxtail millet significantly increased by 9.1 cm and 7.08 cm^2^, while SPAD value increased by 4.3; however, this difference was not significant. After receiving rain for the second time for 5 days, the plant height and SPAD value of foxtail millet in Z2 treatment, as well as the plant height and leaf area of foxtail millet in Z3 treatment were significantly higher than those in CK and the corresponding black film mulching treatments of PZ2 and PZ3. After receiving rain for the third time for 5 days, the plant height, leaf area, and SPAD value of foxtail millet in Z1, Z2, and Z3 treatments were higher than CK and those treated with black film covering PZ1, PZ2, and PZ3 in the corresponding areas; however, the leaf area of foxtail millet treated with Z3 was significantly higher than that of CK (Figure 7, Figure 8 and Figure 9).

#### 2.3.2. Effects of Corn Straw Mulching on Photosynthesis of Foxtail Millet Leaves

**Figure 10:**
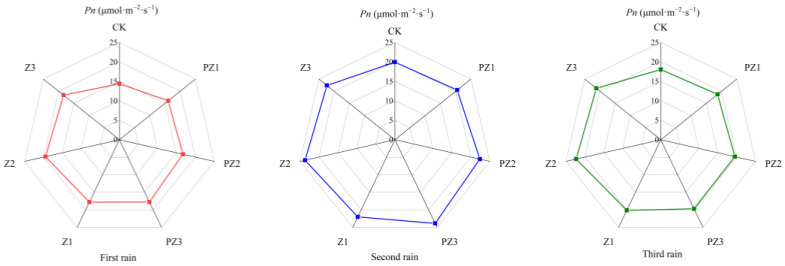
Effect of corn straw mulching on *Pn* of foxtail millet leaves (field experiments).

**Figure 11:**
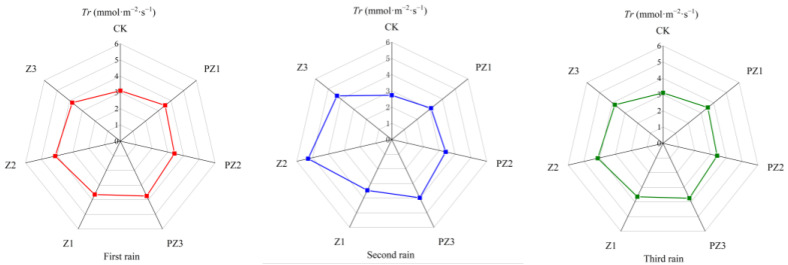
Effect of corn straw mulching on *Tr* of foxtail millet leaves (field experiments).

**Figure 12:**
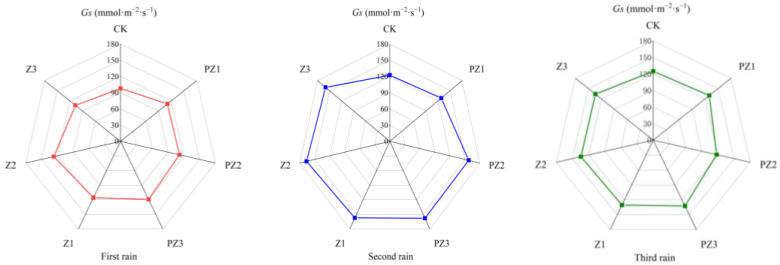
Effect of corn straw mulching on *Gs* of foxtail millet leaves (field experiments).

**Figure 13:**
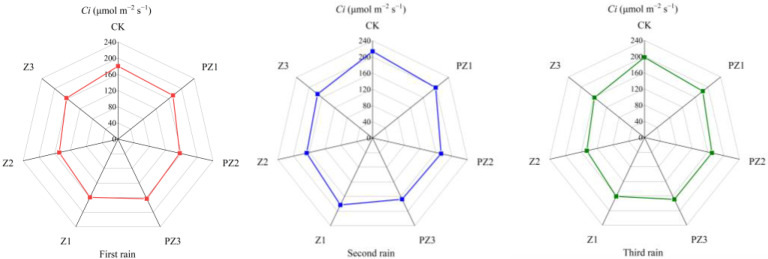
Effect of corn straw mulching on *Ci* of foxtail millet leaves (field experiments).

Adding corn straw to the field can improve the photosynthetic characteristics of foxtail millet. Specifically, the net photosynthetic rate (*Pn*), transpiration rate (*Tr*), and stomatal conductance (*Gs*) of foxtail millet leaves under different mulching amounts were higher than in CK and in the treatments of black film mulching with PZ1, PZ2, and PZ3 in the corresponding areas. However, the intercellular CO_2_ concentration (*Ci*) was lower than in CK and in the treatments of black film mulching with PZ1, PZ2, and PZ3 in the corresponding areas. With the increase in corn straw mulching, the *Pn*, *Tr*, and *Gs* of foxtail millet leaves showed a trend of first increasing and then decreasing, while the *Ci* first decreased and increased. Following 5 days of three spells of rain, the photosynthetic efficiency of foxtail millet leaves was the highest in Z2 treatment. After the first spell of rain for 5 days, compared with CK, the Pn of foxtail millet leaves in Z1 and Z3 treatments was significantly different, which increased by 23.72% and 27.76%, respectively. Compared with PZ1 and PZ3, the *Pn*, *Tr*, *Gs*, and *Ci* of foxtail millet leaves were not significantly different. Compared with CK, the *Pn* and *Tr* of foxtail millet leaves in Z2 treatment were significantly different, increasing by 35.44% and 25.77%, respectively. Compared with PZ2 treatment, the *Pn*, *Tr*, *Gs*, and *Ci* of foxtail millet leaves were not significantly different. After the second spell of rain for 5 days, the transpiration rate of millet leaves treated with Z2 was significantly different from CK and PZ2, and that of foxtail millet leaves treated with Z3 was significantly different from CK, but not significantly different from PZ3. After the 5 days of the third spell of rain, no significant difference was observed in *Pn*, *Tr*, *Gs*, and *Ci* between treatments Z1, Z2, and Z3, as well as CK, and treatments PZ1, PZ2 and PZ3 covered with black film in corresponding areas. Therefore, our findings imply that the application of corn straw mulching and its incorporation into the field primarily impacts the *Pn* and *Tr* of foxtail millet leaves (Figure 10, Figure 11, Figure 12 and Figure 13).

#### 2.3.3. Effects of Corn Straw Mulching on Foxtail Millet Yield

The yield components of foxtail millet were improved by the application of different mulching amounts of corn straw. Specifically, the panicle weight, panicle length, panicle grains weight and the 1000-grain weight of foxtail millet were significantly higher under Z2 and Z3 treatments compared to CK. Compared with the black film mulching treatment in the corresponding area, the panicle length and 1000-grain weight of foxtail millet under Z2 and Z3 treatments were significantly higher than those of foxtail millet under PZ2 and PZ3 treatments, and the nutrients and allelochemicals in corn straw mainly affected the panicle length and 1000-grain weight of foxtail millet. As mulch increased, the yield of foxtail millet first increased and then decreased. The highest yield of foxtail millet under Z2 treatment was 5781.51 kg/ha, which was 10.32% higher than that of CK and 3.96% higher than that under PZ2 treatment. The physical covering effect of corn straw mulching plays a role in saving water and preserving soil moisture. Simultaneously, nutrients and allelochemicals in the straw enter the soil and are absorbed and utilized by foxtail millet, thus increasing the yield of foxtail millet, as indicated by the increasing panicle length and 1000-grain weight of foxtail millet (Table 3).

## 3. Discussion

### 3.1. Allelopathic Effects of Straw Water Extract on Weeds

Crop straw affects seed germination and the seedling growth of plants through allelopathy, but the sensitivity of different plants to allelochemicals varies. Allelochemicals produced by different crop straws have different effects on different plant seeds [34,35], and the intensity of the liquefaction sensitivity of straw extraction in different crops, parts, and concentrations also differs. The water extracts of wheat straw significantly reduced the germination rate, germination index, and all morphological growth parameters of Bermuda grass seedlings [36]. Crude corn straw extract had a greater inhibitory effect on seedling germination and growth than the fermented corn straw extract. The corn straw extract had remarkably inhibited the germination and seedling growth of *C. album* and the seedling growth of *A. theophrasti* [37]. A 40% water extract concentration of *Gaillardia pulchella* can inhibit the germination of *Brassica napus*. Different concentrations of water extracts of *G. pulchella* inhibited the growth of *B. napus* roots but promoted the elongation of shoots [38]. This is similar to the results of this study using corn straw water extract treatments. In this study, the T1 treatment inhibited the germination of the four weeds; reduced the root length, shoot length, and allelopathy index of *S. viridis* and *E. crus-galli*. However, T1 treatment increased the root length, shoot length, and allelopathy index of *C. album*; and reduced the root length of *A. retroflexus*, but increased shoot length. The order of the effects of corn straw water extract from strong to weak on the comprehensive allelopathy index of the four weed seeds was as follows: *C. album* > *S. viridis* > *A. retroflexus* > *E. crus-galli*.

### 3.2. Allelopathic Effects of Corn Straw Mulching on Weeds

Currently, weed control in fields remains an urgent problem, and the growth of weeds affects the growth, yield, and quality of crops. Various herbicides are used on agricultural lands. Long-term use of pesticides causes resistance in weeds and adverse effects, such as soil pollution [39,40]. Black film covering is a common cultivation method in agricultural production, which can increase crop yield, affect crop growth and soil environment [41,42]. Black film covering can weaken the heat exchange between the soil and the outside world, increasing the temperature of the cultivated layer of the soil [43,44]. At the same time, covering with black film can reduce the light under the film, so as to inhibit weed growth. The PZ1, PZ2 and PZ3 treatments in this study only covered the ridges; the plants inside the rows were exposed outside, so the temperature under the film was not too high. Rainfall can seep into the soil from the rows without affecting plant growth and soil moisture loss. Straw covering is a green and environmentally friendly cultivation measure that reduces the occurrence of weeds and environmental degradation, as well as effectively utilizing a renewable resource and promoting the development of green and sustainable agriculture [45]. Corn straw mulching can maintain soil temperature and moisture, it also has an allelopathic effect (promoting and inhibiting) on the germination and growth of plants. Corn straw mulching can increase the root lengths of the deep roots of crops, while the average root length density increases by 11.7% and 15.8% at depths of 30 and 40 cm, respectively [46]. The combination of corn straw mulching and no-tillage maintains soil moisture, initiates the release of available nitrogen, and enhances nitrogen uptake in plants, thereby improving grain yield, water use efficiency, and nitrogen use efficiency [47]. The degradation of straw mulching in soil after corn harvesting can improve the physiochemical properties of the soil, leading to increased water absorption, yield, and water use efficiency in the subsequent generation of corn crops [48]. In this study, the physical covering effect of corn straw mulching saved water, preserved soil moisture, and regulated the soil temperature. At the same time, the allelochemicals in the corn straw had different degrees of allelopathic effects on the weeds and foxtail millet after entering the soil. The results of this experiment showed that the germination and growth of the four weeds were inhibited by the physical covering effect and allelopathy of corn straw mulching. With increased corn straw mulching duration and rainfall, corn straw gradually decomposed and the allelochemicals in the corn straw were gradually diluted, which promoted the germination and growth of weeds, thus gradually reducing their influence on weed density and coverage. The density of the four weeds decreased with an increase in the straw mulching amount and increased with increasing rainfall under different straw mulching amounts.

In this study, the allelopathy of corn straw and its water extracts on four representative weeds in foxtail millet fields was investigated, and the weed species and allelopathy indexes studied were not comprehensive enough.

### 3.3. Effect of Corn Straw Mulching on Foxtail Millet

Straw mulching can effectively utilize soil resources and enhance the vitality of crop roots, thereby promoting the absorption of nutrients and water. This cascade of events enhances the physiological functions of plants and consequently increases crop yield. Intercropping wheat and corn with straw mulching can improve the overcompensation effect of late-maturing corn, ultimately increasing the total yield of the intercropping system [49]. Combining black film with wheat straw mulching increases the leaf area, leaf photosynthetic rate, and the 1000-grain dry weight of summer maize, thereby resulting in an increase in maize yields [50]. Straw mulching alleviated the impact of drought stress on sesame, indicative of the potential of this approach to support sesame yields in arid areas [51]. This study indicates that corn straw mulching improves the plant height, leaf area, SPAD value, leaf photosynthetic efficiency, and yield of foxtail millet. With the increase in straw mulching, the plant height and leaf area of foxtail millet gradually increased, whereas the SPAD value first increased and then decreased. The *Pn*, *Tr*, and *Gs* of foxtail millet leaves show a trend of first increasing and then decreasing with increasing coverage, while the intercellular CO_2_ (*Ci*) concentration first decreased and increased. After covering with corn straw, the panicle length and 1000-grain weight of foxtail millet were increased, resulting in an increased foxtail millet yield.

In this study, the physical covering effect of straw influenced water retention and moisture retention. Additionally, nutrients and allelopathic substances in straw permeated the soil and were absorbed and utilized by foxtail millet, which is also an important driver of crop yield. In future studies, elucidating other indicators is of utmost importance. Meanwhile, further research investigating the effects of corn straw and its water extracts on other weeds and foxtail millet is necessary.

## 4. Materials and Methods

### 4.1. Experimental Materials

Weeds: seeds of four common weeds found in foxtail millet fields were collected from the farming stations: *Chenopodium album* L. (*C. album*), *Setaria viridis* L. (*S. viridis*), *Echinochloa crus-galli* L. (*E. crus-galli*) and *Amaranthus retroflexus* L. (*A. retroflexus*). These weed seeds were collected in the field of foxtail millet at the Agricultural Station of Shanxi Agricultural University in Jinzhong City, Shanxi Province.

Corn straw: corn straw grown in the countryside around Shanxi Agricultural University in Taigu District, Jinzhong City, Shanxi Province was harvested at the maturity stage, dried, and cut into 3 cm pieces, which were stored in a cool and ventilated place for subsequent use.

Foxtail millet variety tested in the field: Zhangzagu 10 (cultivated by the Zhangjiakou Academy of Agricultural Sciences).

### 4.2. Experimental Design

The study was divided into Petri dish and field experiments. All experiments adopted a completely randomized experimental design.

The Petri dish experiment consisted of five treatments: water control (TCK) and four corn straw water extract treatment groups (T1, T2, T3, and T4). The concentrations of T1–T4 corn straw water extracts were 100, 10, 2, and 1 g/L, respectively.

The field experiment consisted of seven treatments: four control treatments, namely, no corn straw (CK), 50% black film (PZ1), 70% black film (PZ2), and 100% black film (PZ3) covering treatments, and three corn straw covering treatments, with coverage amounts of 3000, 6000, and 12,000 kg/ha (Z1, Z2, and Z3, respectively).

#### 4.2.1. Petri Dish Experiment

(1) Preparation of corn straw water extracts:

Air-dried corn straw was crushed using a high-speed crusher and filtered through an 80 mesh sieve to obtain corn straw powder. The corn straw powder was mixed with distilled water in a beaker at a mass ratio of 1:10, placed in an ultrasonic instrument (JP-100S, Jiemeng cleaning equipment Co., LTD., Shenzhen, China) for ultrasonic extraction for 30 min, with the temperature of water at 30 °C. It was centrifuged for 3 min at 6400 rpm, and the supernatant was obtained, which was used as the stock solution of the corn straw water extract (100 g/L). The stock solution of the corn straw extract was diluted with distilled water to obtain 10× (10 g/L), 50× (2 g/L), and 100× (1 g/L) dilutions, which were placed in a refrigerator at 4 °C for subsequent use.

(2) Petri dish weed experiment:

Petri dishes with a diameter of 9 cm were sterilized. After cooling, two layers of sterilized filter paper were placed in each Petri dish, and 5 mL of corn straw water extract stock solution, 10× diluent, 50× diluent, and 100× diluent were added; distilled water was used as a control. Thirty weed seeds were evenly seeded in each Petri dish and placed in a constant temperature incubator at 27 °C and a relative humidity of 50%. After 6 days, the root and shoot lengths of the weed seeds were measured, and each measurement was repeated three times for each treatment. The germination rate, allelopathy index, and comprehensive allelopathy index of the weed seeds were calculated according to the number of germinating seeds observed daily [17]. The calculation formulas used are as follows:

Germination rate = (number of germinations on the 6th day/total number of weed seeds per Petri dish) × 100.

Allelopathy index (RI) = 1-TCK/T or T/TCK-1.

(Where: RI is the allelopathy index, T represents the treatment group data and TCK represents the control group data. When T ≥ TCK, RI = 1-TCK/T, when T < TCK, RI = T/TCK-1)

Comprehensive allelopathy index = the average value of the RI of germination rate, root length and shoot length of weed seeds.

#### 4.2.2. Field Experiment

**Figure 14:**
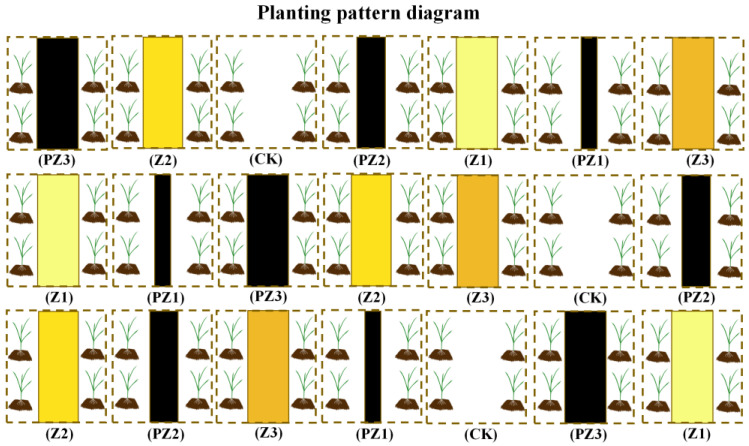
Planting pattern diagram of foxtail millet field.

The experiment was conducted in May 2020 in the experimental base of Shanxi Agricultural University Farming Station. The experiment used a randomized block design (Table 4). Seven treatments were set up in this experiment, each treatment was repeated three times, and the area of each treatment was 30 m^2^ (5 m × 6 m). Soil nutrient content was measured before sowing foxtail millet (Table 5). After the sprouting of foxtail millet, the relevant amounts of corn straw and black film were evenly spread between the rows of foxtail millet in each plot (Figure 14). The black film used in our experiment refers to a black polyethylene plastic film with a thickness of 0.01 mm, provided by Shanxi Yingtai Plastic Co., Ltd. (Yuncheng, China).

Field weed investigation:

In 2020, random quadrat samples were conducted 5 days (29 June, 8 July and 26 July) after each of three rainfall events (Table 6). These samples were conducted in each plot and included weed density, total weed coverage value and weed control effect. The area of the quadrat was 0.25 m^2^ (0.5 m × 0.5 m) and three quadrat areas were investigated for each treatment [11]. The indices were calculated using the following formulas:

Field density (plant/m^2^) = number of weed plants in each survey sample/area.

Coverage (%) = (green vegetation pixels in the sample/total pixels in the survey sample) × 100.

Plant control effect (%) = (number of weeds in the control area − number of weeds in the treatment area/number of weeds in the control area) × 100.

Fresh weight control effect (%) = (weed fresh weight in control area − weed fresh weight in treatment area/weed fresh weight in control area) × 100.

Determining the agronomic traits of foxtail millet:

Five days after three rains in the same year, representative and uniformly growing plants were selected for each treatment. The height of the foxtail millet, as well as the length and width of the second leaf of the plant from top to bottom, were measured using a tape measure [11]. Specifically, the leaf area was determined as follows:

Leaf area (cm^2^) = leaf length × leaf width × 0.75.

Determining the photosynthetic characteristics of foxtail millet:

The SPAD-502 portable chlorophyll analyzer (Minolta Camera Co. Ltd., Osaka, Japan) was used to measure the SPAD values of the second leaf from top to bottom.

On a clear and windless morning (9:00–11:00), photosynthetic parameters including the net photosynthetic rate (*Pn*), transpiration rate (*Tr*), stomatal conductance (*Gs*), intercellular CO_2_ concentration (*Ci*) of the second leaf from top to bottom were measured using a portable handheld photosynthetic instrument CI-340 (CID Bio-Science, Inc., Washington, DC, USA).

Foxtail millet yield:

Before harvesting foxtail millet, we randomly selected panicles to measure phenotypic outcomes such as panicle length, panicle weight, panicle grain weight, and 1000-grain weight. Using the diagonal sampling method, each treatment is 1 m^2^ (1 × 1 m), and then converted into yield.

### 4.3. Data Processing

Data processing was performed using Microsoft Excel 2021 (Microsoft, Redmond, WA, USA) and IBM SPSS Statistics 25 software (SPSS Inc., Chicago, IL, USA). All data analysis was conducted using Duncan’s new multiple range method, including variance and multiple comparisons, with significance at *p* < 0.05. Data were visualized using Origin 2021 software (Originlab, Northampton, MA, USA).

## 5. Conclusions

The seed germination rate, root length, and shoot length of four weeds, *C. album*, *S. viridis*, *E. crus-galli* and *A. retroflexus*, in foxtail millet fields showed obvious allelopathic responses to different concentrations of corn straw water extract during the seed germination period. Among them, 100 g/L corn straw extract had the best inhibitory effect on the seed germination of the four weeds and significantly inhibited the growth of *S. viridis*, *E. crus-galli*, and *A. retroflexus*, but promoted the growth of *C. album*.

For foxtail millet field planting, a suitable amount of corn straw mulching can effectively inhibit the growth of weeds in foxtail millet fields in a short time, reduce the amount of herbicides sprayed, and reduce the environmental pollution caused by pesticides. Here, 3000 kg/ha and 6000 kg/ha corn straw mulching effectively inhibited the occurrence of weeds in the early stage of foxtail millet growth, but this effect weakened with an increase in rainfall. Corn straw mulching at 12,000 kg/ha had the strongest allelopathic inhibitory effect on the four weeds and effectively reduced the presence of *C. album* and *E. crus-galli*.

Corn straw mulching positively impacted the plant height, leaf area, and photosynthetic parameters of Zhangzagu 10, with the net photosynthetic rate and transpiration rate being the most significantly impacted. The yield of foxtail millet was positively impacted under all straw coverage; specifically, its panicle length and 1000-grain weight were improved. The growth parameters of foxtail millet were most positively impacted under corn straw coverages of 6000 and 12,000 kg/ha, respectively, with the growth of foxtail millet indicating that an appropriate amount of corn straw coverage would have an allelopathic effect on the growth of Zhangzagu 10.

Thus, corn straw mulching at 12,000 kg/ha has been identified as the most effective method for controlling malignant weeds in foxtail millet fields and can promote the growth of Zhangzagu 10. The incorporation of crop straw effectively reduces the growth of some weed species. Future studies should focus on investigating the allelopathic effects of corn straw on other weeds and millet varieties. Additionally, determining the appropriate concentrations of corn straw mulch to control weeds in the field is an important aspect that requires further exploration.

## Figures and Tables

**Table 1:** Weed density of four foxtail millet fields under different corn straw mulching (field experiments). “/” indicates that no samples are found in the community.

Rain Frequency	Treatment	*C. album*	*S. viridis*	*E. crus-galli*	*A. retroflexus*
First rain	CK	65.33 ± 7.07 a	26.67 ± 6.11 a	26.67 ± 6.11 a	12.00 ± 2.00 a
	PZ1	41.33 ± 8.86 b	16.00 ± 4.00 ab	16.00 ± 4.00 bc	2.67 ± 0.62 bc
	PZ2	37.33 ± 5.33 b	14.67 ± 2.31 b	21.33 ± 2.31 ab	4.00 ± 1.93 b
	PZ3	/	/	/	/
	Z1	41.33 ± 6.22 b	17.33 ± 6.11 ab	17.33 ± 6.11 b	2.67 ± 0.62 bc
	Z2	29.33 ± 2.31 c	12.00 ± 4.58 b	14.67 ± 3.58 bc	1.33 ± 0.51 c
	Z3	14.67 ± 6.86 d	8.00 ± 2.93 c	9.33 ± 6.93 d	/
Second rain	CK	65.33 ± 8.33 a	10.67 ± 1.11 b	26.67 ± 2.31 a	4.00 ± 2.00 a
	PZ1	29.33 ± 3.24 c	6.67 ± 2.31 c	18.67 ± 2.31 b	2.66 ± 0.61 ab
	PZ2	20.00 ± 2.00 d	8.00 ± 2.00 bc	16.00 ± 4.00 b	1.33 ± 2.30 b
	PZ3	/	/	/	/
	Z1	40.00 ± 4.42 b	24.00 ± 4.00 a	17.33 ± 6.11 b	2.66 ± 0.61 ab
	Z2	38.67 ± 6.07 b	8.00 ± 2.93 bc	16.00 ± 6.93 b	2.66 ± 2.30 ab
	Z3	30.67 ± 4.13 c	/	8.00 ± 6.93 c	1.33 ± 0.50 b
Third rain	CK	80.00 ± 4.33 a	14.67 ± 8.33 b	40.00 ± 6.93 a	4.00 ± 2.00 ab
	PZ1	21.33 ± 6.11 c	9.33 ± 2.31 c	13.33 ± 2.86 d	2.66 ± 0.61 b
	PZ2	20.00 ± 6.93 c	5.33 ± 2.31 d	10.67 ± 4.33 d	2.66 ± 0.61 b
	PZ3	/	/	/	/
	Z1	48.00 ± 6.93 b	26.67 ± 5.73 a	38.67 ± 2.13 a	6.66 ± 2.11 a
	Z2	46.67 ± 4.62 b	17.33 ± 4.04 b	30.67 ± 2.31 b	2.66 ± 0.61 b
	Z3	41.33 ± 8.33 b	16.00 ± 2.05 b	17.33 ± 6.11 c	1.33 ± 0.50 c

Note: Comparison between treatments of different mulching on the same days, with lowercase letters representing a significant difference (*p* < 0.05). The treatments in the table are as follows: no corn straw cover (CK); shading area without corn straw black film, 50% (PZ1), 70% (PZ2), and 100% (PZ3); and corn straw coverage, 3000 kg/ha (Z1), 6000 kg/ha (Z2), and 12,000 kg/ha (Z3).

**Table 2:** Weed control effect under different corn straw mulching (field experiments). “/” indicates that no samples are found in the community.

Rain Frequency	Treatment	Weed Number (Plant)	Plant Control Effect (%)	Fresh Weight (g)	Fresh Weight Control Effect (%)
First rain	CK	130.67 a		125.67 a	
	PZ1	77.33 b	39.49 c	70.00 c	44.21 b
	PZ2	76.00 b	39.29 c	60.67 d	51.49 b
	PZ3	/	/	/	/
	Z1	77.33 b	39.49 c	99.00 b	20.71 d
	Z2	58.67 c	53.92 b	78.00 c	37.99 c
	Z3	32.00 d	74.38 a	49.33 e	61.03 a
Second rain	CK	105.33 a		530.00 a	
	PZ1	45.33 d	56.56 a	363.00 c	32.38 c
	PZ2	58.67 c	44.06 b	299.00 d	44.4 b
	PZ3	/	/	/	/
	Z1	82.67 b	21.56 c	480.67 b	12.30 d
	Z2	62.67 c	40.50 b	354.67 c	33.44 c
	Z3	38.67 d	62.76 a	211.00 e	60.11 a
Third rain	CK	134.67 a		827.00 a	
	PZ1	44.00 e	64.46 ab	797.33 a	3.37 c
	PZ2	36.00 f	71.22 a	752.67 b	11.28 a
	PZ3	/	/	/	/
	Z1	94.67 b	23.44 d	800.67 a	3.18 c
	Z2	81.33 c	36.43 c	759.00 b	8.22 b
	Z3	54.67 d	60.09 b	733.33 c	8.87 b

Note: Comparison between treatments of different mulching on the same days, with lowercase letters representing a significant difference (*p* < 0.05).

**Table 3:** The yield components of foxtail millet (field experiments).

Treatments	Panicle Weight (g)	Panicle Length (cm)	Panicle Grains Weight (g)	1000-Grain Weight (g)	Yield (kg/ha)
CK	18.57 ± 1.002 c	26.10 ± 2.61 c	11.51 ± 0.64 c	2.96 ± 0.01 b	5240.88 ± 159.95 c
PZ1	18.66 ± 0.50 c	27.23 ± 0.47 bc	11.99 ± 1.33 bc	2.96 ± 0.04 b	5325.65 ± 226.36 c
PZ2	19.23 ± 1.36 bc	28.07 ± 0.78 bc	12.18 ± 0.98 bc	2.97 ± 0.03 b	5561.12 ± 157.80 b
PZ3	20.81 ± 0.40 ab	28.63 ± 0.55 b	13.75 ± 0.70 bc	2.98 ± 0.04 b	5666.78 ± 136.96 ab
Z1	18.78 ± 1.73 c	29.23 ± 1.06 b	12.56 ± 2.73 bc	3.02 ± 0.01 ab	5681.40 ± 209.57 ab
Z2	22.68 ± 1.76 a	34.03 ± 0.59 a	15.48 ± 1.22 a	3.07 ± 0.05 a	5781.51 ± 333.98 a
Z3	21.29 ± 0.05 a	32.23 ± 1.36 a	14.46 ± 1.24 ab	3.07 ± 0.02 a	5711.02 ± 336.76 a

Note: Comparison between treatments of different mulching on the same days, with lowercase letters representing a significant difference (*p* < 0.05).

**Table 4:** Treatment conditions of field experiment.

Treatment	CK	PZ1	PZ2	PZ3	Z1	Z2	Z3
Corn straw mulching amount (kg/ha)	0	0	0	0	3000	6000	12,000
Black film covering area (%)	0	50%	70%	100%	0	0	0

**Table 5:** Nutrient content of foxtail millet field soil.

Year	Soil Depth (cm)	pH	Available K (mg/kg)	Available P (mg/kg)	Available N (mg/kg)	Total N (g/kg)	Total P (g/kg)	Total K (g/kg)	Organic (g/kg)
2019	0~5	8.21	484.20	43.70	64.20	1.22	0.76	19.36	8.52
	5~10	8.14	471.90	26.79	42.80	1.27	0.76	18.08	8.52
	10~15	8.17	319.60	21.28	49.93	1.11	0.77	18.53	7.99
2020	0~5	8.11	377.40	21.06	48.20	1.11	0.91	21.91	8.09
	5~10	8.19	297.80	18.93	56.55	1.04	0.81	23.33	8.52
	10~15	8.10	303.90	24.10	51.77	1.07	0.87	21.76	8.22

**Table 6:** Rain events in 2020.

Rainfall Frequency	Rainfall Date	Rainfall Time	Precipitation (mm)	Measurement Date
First rain	29 June	8:40 a.m. to 11:30 a.m.	18	4 July
Second rain	8 July	6:00 a.m. to 2:30 p.m.	52.3	13 July
Third rain	26 July	11:30 a.m. to 5:20 p.m.	43.9	31 July

## Data Availability

The data that support this study are available upon reasonable request from the corresponding author.
